# The Predictive Value of Serum Cytokines for Distinguishing Celiac Disease from Non-Celiac Gluten Sensitivity and Healthy Subjects

**DOI:** 10.29252/ibj.24.6.335

**Published:** 2020-06-01

**Authors:** Fatemeh Masaebi, Mehdi Azizmohammad Looha, Mohammad Rostami-Nejad, Mohamad Amin Pourhoseingholi, Navid Mohseni, Gabriel Samasca, Iulia Lupan, Mostafa Rezaei-Tavirani, Mohammad Reza Zali

**Affiliations:** 1 Depatment of Biostatistics, Faculty of Paramedical Sciences, Shahid Beheshti University of Medical Sciences, Tehran, Iran;; 2 Gastroenterology and Liver Diseases Research Center, Research Institute for Gastroenterology and Liver Diseases, Shahid Beheshti University of Medical Sciences, Tehran, Iran;; 3 *Department of Immunology, Iuliu Hatieganu University of Medicine and Pharmacy ClujNapoca, Romania* *; *; 4 Department of Molecular Biology and Biotechnology, BabesBolyai University, Cluj-Napoca, Romania;; 5 Proteomics Research Center, Faculty of Paramedical Sciences, Shahid Beheshti University of Medical Sciences, Tehran, Iran

**Keywords:** Celiac disease, Cytokines, Sensitivity and specificity

## Abstract

**Background:**

It has been established that the level of some inflammatory cytokines increases in CD and NCGS in comparison with healthy subjects. Therefore, the primary interest in our research was proposing an accurate tool to diagnose patients with CD and NCGS from healthy individuals in an Iranian population.

**Methods:**

The serum samples were examined in 171 participants, including 110 CD patients, 46 healthy individuals, and 15 NCGS. The commercial ELISA kits were used to detect the level of the following cytokines: IL-1, IL-6, IL-8, IL-15, and IFN-γ. The ROC curve analysis was applied to determine the optimal thresholds for high sensitivity, specificity, positive and negative predictive values of cytokines, as the indicators of CD, NCGS, and healthy control groups.

**Results:**

In NCGS group, the values of AUC for IL-1, IL-8, and IFN-γ were 71%, 78%, and 70%, respectively. To differentiate the CD and NCGS groups from the control group, IL-15 had the highest sensitivity (82.70%), specificity (56.50%), positive predictive value (81.98%), and negative predictive value (57.78%), followed by IL-8 with the highest sensitivity of 74.50%, specificity of 73.30%, and positive and negative predictive values of 95.35% and 30.21%, respectively.

**Conclusion:**

The obtained results demonstrate that IL-15 and IL-8 could be proposed as potential markers in their optimal cut-off points for distinguishing CD from the NCGS and the healthy control. Based on our findings, the evaluation of cytokine levels can be recommended as a useful tool for the diagnosis of CD and NCGS in a clinical practice.

## INTRODUCTION

Celiac disease is an autoimmune *disorder *occurred in genetically predisposed people whose small intestine is unable to properly absorb nutrients from food^[^^1^^]^. Gluten consumption by CD patients triggers an immune response in their small intestine. Gluten is a group of proteins, mainly found in wheat, rye, and barley, as well as in products derived from these grains. *CD* is a major public health concern all over the world and affects approximately 1% of the population of South America, Africa, Europe, and Asia^[^^2^^]^. This rate varies from 0.5% to 1.0% for Iranian population^[^^3^^]^. 

CD diagnosis is based on serological assays (endomysial antibody, deamidated gliadin peptide, and tTG), and patients with positive results are examined using endoscopy and small intestinal biopsies^[^^4^^]^. *It is widely accepted* that CD is a T cell-mediated disease that modifies gliadin-derived peptides represented by HLA-DQ2 and HLA-DQ8^[^^4^^]^. The enzymatic activities of tTG can result in the activation of autoreactive T-helper cells, followed by the production of proinflammatory cytokines and intestinal inflammation^[^^5^^]^. 

Proinflammatory cytokines such as IFN-γ and IL-15 play critical roles in CD pathogenesis as the *elevated levels* of these *cytokines* are *related* to the severity of the disease^[^^5^^]^. Meanwhile, production of IFN-γ leads to T cells activation in small intestinal mucosa, which observed in the active form of CD^[^^6^^]^. Activation of intestinal intraepithelial lymphocytes is main cause of *IL-15 elevation*^[^^7^^]^. *The role of proinflammatory* cytokines such as *IL-1β and* TNF-α *in mediating the mucosal damage* has also been investigated. *High amount of IL-1β and* TNF-α results in overexpression of *specific* matrix *metalloproteinases* 1 and 3^[^^5^^,^^8^^]^. On the other hand, those subjects who are negative for CD and responded to the gluten-free diet are classified as having *NCGS*^[^^9^^,^^10^^]^. Diagnosis of NCGS remains as a controversial challenge, because there are no reliable diagnostic markers^[^^11^^,^^12^^]^. Since HLA-DQ2/DQ8 is present only in the minority of patients with NCGS, it has insufficient sensitivity for diagnosis of NCGS^[^^13^^,^^14^^]^. It has been reported that CD and NCGS patients have different reactions to *gluten ingestion. CD* patients *have *both *innate* and *adaptive immune* responses, but only innate immunity may be activated in NCGS patients^[^^15^^]^. Therefore, in both patient groups, the level of IL-1β, IL-6, IL-8, IFN-γ, IL-2, and IL-15 may be determining factors^[^^16^^]^. 

At the systemic level, a few studies have measured the *serum levels* of *cytokines, using* ELISA for CD patients^[^^5^^,^^17^^]^. Findings have indicated that the elevated levels of IL2, IL-18, and IFN-γ increase the chance of active CD^[^^18^^-^^20^^]^. As mentioned above, the inflammatory immune responses raised by the ingestion of gluten are *involved in both* CD and NCGS patients^[^^15^^]^; hence, it might be necessary to assess the diagnostic and predictive value of inflammatory cytokines as a tool for discrimination between CD and NCGS patients. The goal of the present study was comparing and evaluating the sensitivity, specificity, and predictive power of cytokines IL-6, IL-1, IL-15, IL8, and IFN-γ by using AUC criteria in two subgroups, first by comparing CD with NCGS and then by comparing CD with healthy group.

## MATERIALS AND METHODS

**Study population**

In January 2016, this study was conducted at the Research Institute for Gastroenterology and Liver Diseases at Shahid Beheshti University of Medical Sciences as the main referral center for CD patients in Tehran, Iran. The diagnosis of CD patients was performed based on the serological tests such as anti-tTG antibodies and endoscopy. Seropositive patients underwent endoscopy; however, with respect to the Marsh classification, those with the histopathological changes of small intestine were confirmed as CD patients. Serum samples were collected from 110 CD patients. The gastrointestinal symptoms of patients were also collected immediately at the time of diagnosis. In addition, 46 subjects without any disease were included as control. Finally, 15 NCGS patients with negative serologic tests results for CD and wheat allergy were included in this study, but the *patients with the history of other autoimmune disorders such as inflammatory bowel disease and Type 1 diabetes were excluded*. 

**Sample collection**

Peripheral blood samples (10 ml) were obtained from each CD and NCGS patients and also healthy controls, and then serum was stored at -80 °C immediately after taking the blood sample. Moreover, the characteristics and medical history of the participants were recorded by questionnaires.

**Measurement of cytokine levels**

The commercial *ELISA* kits (eBioscience, USA) were used to quantify the IL-1, IL-6, IL-8, IL-15  and IFN-γ levels, according to the manufacturer’*s standard protocol. *Serum or plasma samples (50 µl) were used in these assays. The sensitivity and the brands of ELISA kits for IL-15, IL-8, IL-1, IL-6,  and IFN-γ were as 3.4 pg/mL (Invitrogen™ BMS2106), 2.0 pg/mL (Invitrogen™ BMS204-3TENCE), 0.3 pg/mL (Invitrogen™ BMS224-2), 0.01 ng/mL (Invitrogen™ BMS214TEN), and 0.99 pg/mL (Invitrogen™ BMS228CE). The sensitivity and specificity are statistical measures that refer to the ability of the test to correctly identify an individual as disease (CD) and disease-free (NCGS or control group), respectively. Positive predictive value and negative predictive value are the probabilities explaining that individuals with positive and negative clinical tests are truly disease and disease-free, respectively^[^^21^^]^. 

**Table 1:** D*escription* of cytokines in CD, NCGS, and control groups

Cytokines	**Examined group**	**No. of patients **	**Mean (ng/ml)**	**SD**
**IL-1**	CD	110	5.12	9.86
	NCGS	15	6.56	18.51
	**Control **	46	2.63	1.65
				
IL-6	CD	110	9.79	7.11
	NCGS	15	8.38	5.90
	**Control **	46	11.17	10.18
				
IL-8	CD	110	115.69	128.06
	NCGS	15	40.18	43.65
	**Control **	46	81.00	81.42
				
IL-15	CD	110	219.57	370.14
	NCGS	15	96.33	54.37
	**Control **	46	143.57	226.43
				
IFN-γ	CD	110	76.27	132.92
	NCGS	15	26.33	59.25
	**Control **	46	42.41	82.93

**Statistical analysis**

In this study, *ROC* curve analysis and *Youden’s Index* were employed to determine the *optimal* *thresholds* for detecting the high sensitivity, specificity, positive and negative predictive values of cytokines IL-1, IL-6, IL-8, IL-15, and IFN-γ. The mentioned cytokines were used as an accuracy diagnostic tool for distinguishing CD patients from NCGS and also from healthy groups. Moreover, the simultaneous effect of cytokines for the detection of CD patients was investigated using the logistic regression model. *Statistical analyses* were *performed using IBM SPSS Statistics* for Windows, *Version* 25.0. (IBM Corp., Armonk, NY, USA) and R 3.4.1 software.

**Ethical statement**

The above-mentioned sampling protocols were approved by the Medical Ethics Committee of the Research Institute for Gastroenterology and Liver Diseases, Shahid Beheshti University of Medical Sciences, Tehran, Tehran province, Iran (ethical code: IR.SBMU.RETECH.REC.1397.1013). Written informed consents were provided by the patients. 

## RESULTS

**Fig. 1:**
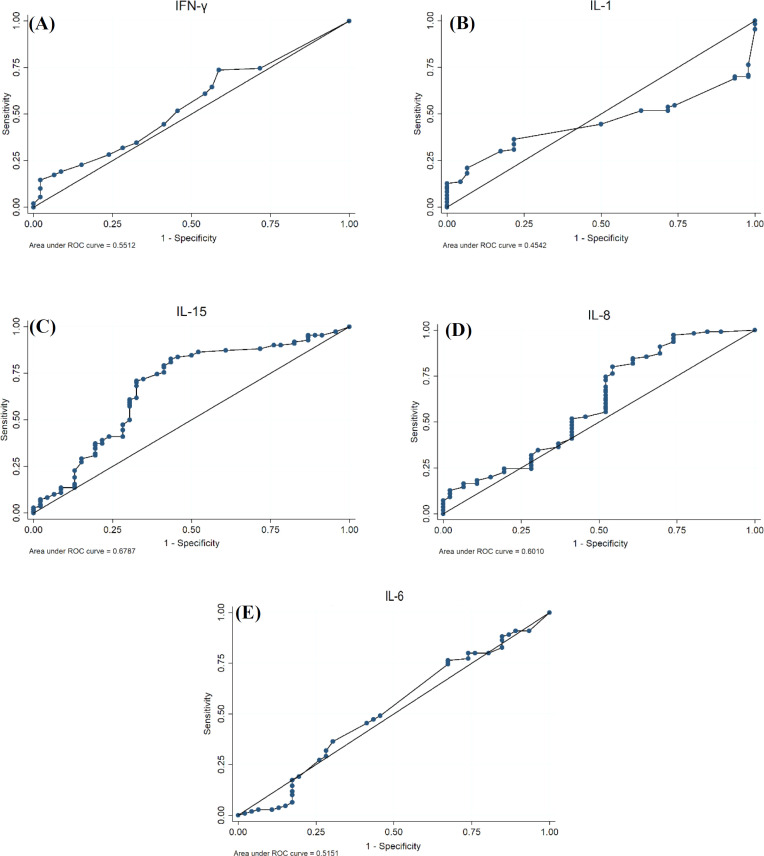
ROC curve for CD patients and healthy controls showing the plot and the best cut-off point for (A) IFN-γ (B) IL-1, (C) IL-15, (D) IL-8, and (E) IL-6

**Fig. 2:**
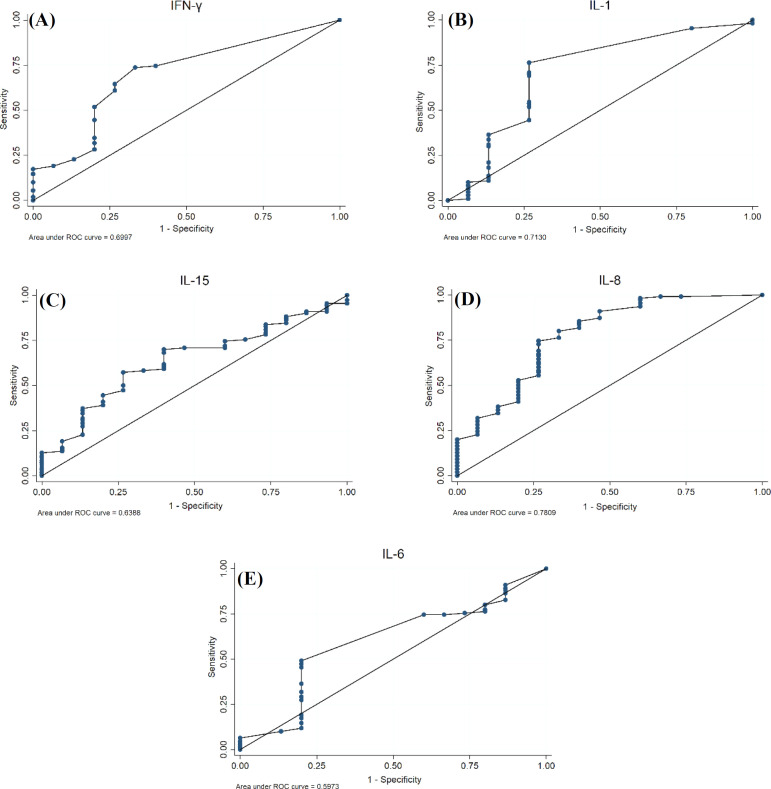
ROC curve for CD patients and NCGS patients showing the plot and the best cut-off point for (A) IFN-γ (B) IL-1, (C) IL-15, (D) IL-8, and (E) IL-6

A total of 110 patients with CD (with the mean age of 33.6 ± 11.87 years old; 34.5% of them were male), 15 patients with NCGS (with the mean age of 38.7% ± 13.75 years old; 46.7% of them were male), and 46 healthy individuals (with the mean age of 38.8 ± 13.74 years old; 43.4% of them were male) were enrolled in this study. The *description* of cytokines in each group is shown in Table 1. As shown in Table 2, the best cut-off points for the IL-1, IL-6, IL-8, IL-15, and IFN-γ for the diagnosis of CD patients in the control group were 2.34 ng/mL, 5.80 ng/mL, 26.66 ng/mL, 68.79 ng/mL, 1.50 ng/mL, and for diagnosis of CD patients in NCGS group were 1.07 ng/mL, 6.25 ng/mL, 29.51 ng/mL, 86.17 ng/mL, and 1.50 ng/ml, respectively. Besides, in the CD-NCGS group, none of the AUC values were greater than 70% except those for IL-1 and IL-8. The results of the *ROC* analysis are shown in Table 2, and the ROC curve for each cytokine and their combination was made to compare the CD with the control group and also the CD with NCGS group (Figs. 1 and 2).

**Table 2:** ROC analysis of different cytokines for the diagnosis of CD in control and NCGS groups

**Group**	**Cytokines**	**Cut-off point (ng/ml)**	**AUC**	**Sensitivity**	**Specificity**	**PPV**	**NPV**
CD vs. control	IFN-γ	1.50	0.55	73.60	41.30	73.20	33.90
	IL-1	2.34	0.45	36.40	78.30	80.00	33.96
	IL-15	68.79	0.68	82.70	56.50	81.98	57.78
	IL-6	5.80	0.51	76.40	32.60	73.04	36.59
	IL-8	26.66	0.60	80.00	45.70	77.88	48.84
	Total^*^	0.29	0.66	44.5	87.0	61.90	19.44
CD vs. NCGS	IFN-γ	1.50	0.70	73.60	66.70	94.19	25.64
	IL-1	1.07	0.71	95.45	40.30	89.43	25.50
	IL-15	86.17	0.64	57.30	73.30	94.03	18.97
	IL-6	6.25	0.59	49.10	80.00	94.74	17.65
	IL-8	29.51	0.78	74.50	73.36	95.35	30.21
	Total	0.13	0.75	70.2	60.0	76.60	11.22

PPV, positive predictive value; NPV, negative predictive value

## DISCUSSION

In this study, for the ﬁrst time, the possible diagnostic performance of cytokines for CD and NCGS were evaluated. Our findings demonstrated that IL-8 and IL-15 had the highest sensitivities, specificities, positive and negative predictive values for the detection of the CD patients compared to the NCGS group and healthy control. Also, the combination of all investigated cytokines *in the *CD group indicated no significant effect on the diagnosis of CD patients. The pleiotropic cytokine *IL*-*15, as a protein* with a wide variety of biological functions, has 114 amino acids, which were primarily produced by mononuclear phagocytes in stressful conditions^[^^22^^]^. This cytokine plays a key role in the development of organ-specific autoimmune and inflammatory diseases^[^^23^^]^. Many studies have considered its performance on the pathogenesis of CD and also confirmed its effects on immunological responses^[^^22^^]^. In a study conducted by Di Sabatino *et al.*^[^^24^^]^, 46 CD patients and 22 healthy individuals were evaluated, and their results indicated that the expression of IL-15 level increased in the intestine of CD patients compared to the healthy group, which means that the lower immunological threshold of IL-15 in CD results in the development of other immune responses and small bowel lesion. Bernardo *et al.*^[^^25^^]^ also obtained the same result after the evaluation of 42 CD patients and 24 healthy individuals in a gastroenterology clinic for intestinal pathologies. However, IL-15 did not increase in NCGS group^[^^7^^]^. In our study, the diagnostic power of the IL-15 was evaluated and indicated that it is not an efficient tool for detecting CD (sensitivity = 57.30%) in the NCGS group. Furthermore, more than 68.79 ng/ml of this cytokine could be considered as an evidence of CD by comparing it with the healthy control group. Heydari *et al.*^[^^7^^]^ and Di Sabatini *et al.*^[^^24^^]^ reported that the *serum* IL-1α and IL-1β *levels were not* *significantly* different among CD, NCGS, and healthy control groups. In contrast, Manavalan *et al.*^[^^5^^]^ showed a high level of IL-1β in CD patients compared to the control group. Based on the different results of past studies, we examined the accuracy of this cytokine as a diagnostic tool for CD patients. Finally, it resulted in 95.45% sensitivity for the detection of CD patients against NCGS group, which makes it an appropriate tool for detecting CD.

IL-8, as a proinﬂammatory chemokine, belongs to chemokine family and plays a pivotal role in *neutrophil degranulation. In *previous studies, a significant expression of IL-8 was demonstrated in the CD patients compared to other groups, which could be explained as the long time activation of proinflammatory responses in the intestine of CD patients^[^^7^^,^^26^^]^. In addition, IL-8 (>29.5) almost had a high accuracy (78%) to diagnose CD patients among NCGS population.

IL-6, a pleiotropic cytokine, is mainly produced in lamina propria myeloid cells in response to intestinal damage and has a significant function in inflammation, as well as in *mediating* *the* *innate* and *adaptive* immune *responses, making *IL-6 an important factor in CD pathogenesis^[^^7^^,^^27^^]^. The result of a number of previous studies showed the highest level of IL-6 in the serum of CD patients in comparison with the NCGS and control group^[^^5^^,^^7^^,^^24^^]^. In NCGS, the low level of IL-6 was reported, and its increased level was not sufficiently *accurate* for *detecting* CD patients in this study population^[^^28^^]^.

IFN-γ, an important mediator of immunity and inflammation, is expressed by *Th1* cells. This critical cytokine plays an *essential* role in *both* *innate and adaptive* *immune systems. *Manavalan *et al.*^[^^5^^]^ reported an elevation in the levels of IFN-γ in CD group. Many other studies have also shown that the increased expression level of IFN-γ in lamina propria may lead to histological changes in the small *bowel of* *CD* patients^[^^5^^,^^29^^,^^30^^]^. In a case-control study on 50 Iraqi CD patients, Abed *et al.*^[^^30^^]^ indicated a higher level of IFN-γ in CD group in comparison with control subjects. In our study, the increased production of IFN-γ cytokine in active CD is in contrast with low levels of IFN-γ in the NCGS group. Di Sabatino *et al.*^[^^24^^]^ and Losurdo *et al.*^[^^31^^]^ have also shown that the expression of IFN-γ in CD was significantly higher than both control and NCGS subjects. These results are completely consistent with our research results. As in our study, when the level of IFN-γ is higher than 1.50 ng/ml, it has an acceptable accuracy and can be used as a tool to detect CD patients among NCGS group. These results are completely consistent with our research results. 

This study indicated that the serum levels of IL-8 and IL-15 in their best cut-off points were potential biomarkers for distinguish CD patients from NCGS and control groups. Moreover, the simultaneous increment of these five cytokines (IL-15, IL-8, IL-1, IL-6, and IFN-γ) provided 75% diagnostic accuracy in distinguishing the NCGS groups from CD patients. Although these tests cannot be used for measuring the systemic inflammation at this time, our findings propose the use of cytokines assay for the diagnosis of CD and NCGS in a clinical practice.

## CONFLICT OF INTEREST.

None declared.
